# Adaptive Optimal Control of Hybrid Electric Vehicle Power Battery via Policy Learning

**DOI:** 10.1155/2023/8288527

**Published:** 2023-05-29

**Authors:** Qinglin Zhu, Huanli Sun, Ziliang Zhao, Yixin Liu, Jun Zhao

**Affiliations:** ^1^College of Transportation, Shandong University of Science and Technology, Qingdao 266590, China; ^2^China FAW Group Corporation, Changchun 130011, China; ^3^College of Mechanical and Electronic Engineering, Shandong University of Science and Technology, Qingdao 266590, China

## Abstract

An online policy learning algorithm is used to solve the optimal control problem of the power battery state of charge (SOC) observer for the first time. The design of adaptive neural network (NN) optimal control is studied for the nonlinear power battery system based on a second-order (RC) equivalent circuit model. First, the unknown uncertainties of the system are approximated by NN, and a time-varying gain nonlinear state observer is designed to address the problem that the resistance capacitance voltage and SOC of the battery cannot be measured. Then, to realize the optimal control, a policy learning-based online algorithm is designed, where only the critic NN is required and the actor NN widely used in most design of the optimal control methods is removed. Finally, the effectiveness of the optimal control theory is verified by simulation.

## 1. Introduction

Nowadays, electric vehicles are developing at a high speed [1]. The power battery provides the required high power for vehicle start stop, acceleration and deceleration, and other instabilities and greatly improves the service life of fuel cells by controlling the charging and discharging power of the power battery [1, 2]. As an important energy storage part of fuel-cell hybrid vehicles, it has far-reaching significance for the research of power cells. The state of charge (SOC) in the battery is one of the important parameters of the battery management system (BMS), but SOC cannot be directly measured by the on-board sensors. Therefore, SOC estimation is a very important problem in the theory and application. Moreover, the power battery is a highly complex nonlinear system in its working state, which greatly increases the difficulty of estimation [3].

In order to meet the requirements of accurate, fast, and real-time estimation of power battery SOC under different conditions, scholars have carried out a lot of advanced achievements. In [4], the authors proposed an observer-based unilateral Lipschitz conditional nonlinear system control method for a class of nonlinear systems with time-varying parameter uncertainties and norm bounded disturbances. For the state-space equation of the equivalent circuit model, a power battery SOC estimation method based on nonlinear observer is proposed in [5]. The authors in [6] introduced the second-order resistance capacitance (RC) model of the battery pack. Under the unilateral Lipschitz condition, a nonlinear observer based on the H∞ method is designed, but whether the optimal performance of the observer can be guaranteed remains to be verified. For the problem of optimal control design of the observers, the authors proposed an adaptive neural network backstepping recursive optimal control method for nonlinear strict feedback systems with state constraints [7]. The neural network (NN) state identification is used to approximate the unknown nonlinear dynamics, and under the actor-critic structure, the virtual and actual optimal controllers are constructed through the backstepping recursive control algorithm. Because actor-critic structure-based adaptive laws are generated on the basis of the square of Behrman residual error obtained by the gradient descent method, these methods are too complex and difficult to implement. In this regard, the authors in [8] proposed an optimal control method based on reinforcement learning (RL) for a class of nonlinear strict feedback systems with unknown dynamic functions. This method eliminates the persistent excitation assumption necessary for most RL-based adaptive optimal control. On this basis, the adaptive NN output-feedback optimal control problem for a class of strict feedback nonlinear systems with unknown internal dynamics, input saturation, and state constraints is studied in [9]. In [10, 11], the authors proposed the novel optimal control algorithm based on advanced AI techniques, which further promotes the development of the optimal control theory.

Inspired by the abovementioned research results, a nonlinear observer with time-varying gain is designed in this paper. Based on the unilateral Lipschitz condition, the nonlinear dynamic problem contained in the system output is solved. The internal unknown dynamic function is approximated by NN to estimate the SOC and the resistance capacitance voltage of the dynamic battery in the power system. Then, based on estimated system states, we develop a policy learning-based optimal control and the estimated weight error is convergence to zero. Finally, the simulation results show the effectiveness of the proposed method.

The innovations of this paper are summarized as follows:

1. The optimal control method based on critic NN is used to solve the optimal control problem of the power battery SOC observer for the first time.
2. Only one critic NN is used to ensure the convergence of the NN weights; thus, the actor NN widely used in most design of optimal control methods [12–14] is removed.
3. Unlike the existing optimal control with known state, the battery state in this paper is unknown. This leads to a complex optimal control problem.

## 2. System Modeling

**Figure 1:**
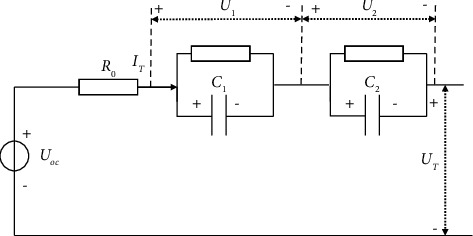
The schematic diagram of the second-order RC model.

In this paper, we consider the second-order RC equivalent circuit model as shown in Figure 1 [15], where *U*_oc_ is the open-circuit voltage (OCV) respected to SOC, *I*_*T*_ represents the current, *U*_*T*_ denotes the terminal voltage, *R*_0_ is the ohmic resistance, *R*_1_ and *R*_2_ are the electrochemical polarization resistance and the concentration polarization resistance, respectively, and *C*_1_ and *C*_2_ are the capacitances. *U*_1_ and *U*_2_ show the voltage of the electrochemical capacitor *C*_1_ and concentration polarization capacitor *C*_2_, respectively.

Then, based on the Kirchhoff voltage laws, the state equation of Figure 1 can be given as

(1) $$\begin{array}{c} \left\{\begin{array}{c} {\dot{U}}_{1}=-\frac{1}{R_{1}C_{1}}U_{1}+\frac{1}{C_{1}}I_{T},\\ {\dot{U}}_{2}=-\frac{1}{R_{2}C_{2}}U_{2}+\frac{1}{C_{2}}I_{T},\\ S\dot{O}C=-\frac{1}{Q_{n}}I_{T}\text{,} \end{array}\right. \end{array}$$

where *Q*_*n*_ is the nominal capacity of the battery.

Then, its output equation can be defined as

(2) $$\begin{array}{c} U_{T}=U_{oc}\left(SOC\right)-R_{0}I_{T}-U_{1}-U_{2}, \end{array}$$

where 0 ≤ SOC ≤ 1, and *U*_oc_(SOC) is the nonlinear monotone increasing function.

Based on (1) and (2), we can obtain state space equation as follows:

(3) $$\begin{array}{c} \left\{\begin{array}{c} \dot{x}=Ax+Bu,x\left(0\right)=x_{0},\\ y=g\left(x\right)+Cx-R_{0}u, \end{array}\right. \end{array}$$

where $x={\left[\begin{array}{ccc} U_{1} & U_{2} & SOC \end{array}\right]}^{T}\in {\mathbb{R}}^{3}$, *y*=*U*_*T*_ ∈ *ℝ*, *u*=*I*_*T*_ ∈ *ℝ*, *g*(*x*)=*U*_*oc*_(SOC) ∈ *ℝ*, and *x*_0_ is the initial state.

(4) $$\begin{array}{c} A=\left[\begin{array}{ccc} -\frac{1}{R_{1}C_{1}} & 0 & 0\\ 0 & -\frac{1}{R_{2}C_{2}} & 0\\ 0 & 0 & 0 \end{array}\right]\in R^{3\times 3},B={\left[\begin{array}{ccc} \frac{1}{C_{1}} & \frac{1}{C_{2}} & -\frac{1}{Q_{n}} \end{array}\right]}^{T}\in R^{3},\\ C=\left[\begin{array}{ccc} -1 & -1 & 0 \end{array}\right]\in R^{1\times 3}\text{.} \end{array}$$

As the power battery is a highly complex nonlinear system in its working state, there are many unknown uncertainties such as ambient temperature, battery self-discharge, battery life, and cycle interval. Therefore, the state space expression (3) can be expressed as follows:

(5) $$\begin{array}{c} \left\{\begin{array}{c} \dot{x}=Ax+Bu+d\left(x\right),x\left(0\right)=x_{0},\\ y=g\left(x\right)+Cx-R_{0}u, \end{array}\right. \end{array}$$

where *d*(*x*) represents nonlinear characteristics.

**Assumption 1 .**

In this paper, we assume that (*A*, *B*) is stabilizable and (*A*, *C*) is detectable. The nonlinear term *d*(*x*) is continuous and bounded.

Control objective: for the second-order RC equivalent model of power battery, based on an adaptive observer a policy learning algorithm-based optimal controller is designed to guarantee all signals of the closed-loop system uniformly ultimately bounded (UUB).

According to the second-order RC model of the power battery, we can derive its state space (3) or (5); then, we should design the control law *u* for the derived state space equation. Thus, we will use the NN observer and the policy learning algorithm to design the control law *u.*

## 3. Optimal Control of Power Battery

### 3.1. Observer Design via NN

This section will design an observer to estimate the battery voltage and SOC. Thus, we assume

(6) $$\begin{array}{c} d\left(x\right)=W_{1}^{T}\sigma \left(x\right)+\varepsilon \left(x\right)\text{,} \end{array}$$

where *W*_1_ ∈ *ℝ*^*N*^ is the ideal NN weights, *σ*(*x*) ∈ *ℝ*^*n*^⟶*ℝ*^*N*^ is the activation function, and *ε*(*x*) ∈ *ℝ* denotes the NN error.

In this paper, the function *d*(*x*) is unknown continuous; hence, the estimated function is

(7) $$\begin{array}{c} \hat{d}\left(x\right)={\hat{W}}_{1}^{T}\sigma \left(x\right)\text{,} \end{array}$$

where ${\hat{W}}_{1}$ is the estimation of *W*_1_.

Then, based on (5) and (7), the observer can be designed as

(8) $$\begin{array}{c} \left\{\begin{array}{c} \dot{\hat{x}}=A\hat{x}+Bu+{\hat{W}}_{1}^{T}\sigma \left(\hat{x}\right)+L{\left[\frac{\partial g}{\partial x}\right]}_{x=\hat{x}}^{T}\left(y-\hat{y}\right),\\ \hat{y}=C\hat{x}+g\left(\hat{x}\right)-R_{0}u\text{,} \end{array}\right. \end{array}$$

where $\hat{x}$ is the estimation of *x*, *L*=*P*^−1^ ∈ *ℝ*^3×3^ is the observation matrix, *P* is the positive matrix, and $\hat{y}$ is the estimation of *y*.

We define the observation error

(9) $$\begin{array}{c} \tilde{x}=x-\hat{x}\text{.} \end{array}$$

Then, from (5) and (8), we can obtain the observation error dynamic equation as

(10) $$\begin{array}{c} \dot{\tilde{x}}=\left[A-L{\left(\frac{\partial g}{\partial x}\right)}^{T}C\right]\tilde{x}-L{\left(\frac{\partial g}{\partial x}\right)}_{x=\hat{x}}^{T}\tilde{g}+W_{1}^{T}\left(\sigma \left(x\right)-\sigma \left(\hat{x}\right)\right)+{\tilde{W}}_{1}\sigma \left(\hat{x}\right)+\varepsilon \text{,} \end{array}$$

where $\tilde{g}=g\left(x\right)-g\left(\hat{x}\right)=\partial g/\partial x\left|x=\xi \left(x-\hat{x}\right)\right.,{\tilde{W}}_{1}={\hat{W}}_{1}-W_{1}$ is the NN weight error.

**Lemma 2 .**

For system (5), if it adopts designed observer (8), the NN weights ${\hat{W}}_{1}$ satisfy the adaptive law

(11) $$\begin{array}{c} {\dot{\hat{W}}}_{1}=-\sigma \left(\hat{x}\right){\tilde{x}}^{T}P\text{.} \end{array}$$

This can guarantee that errors $\tilde{x}$ and ${\tilde{W}}_{1}$ are UUB.

**Proof**

Consider a Lyapunov function

(12) $$\begin{array}{c} V_{1}=\frac{1}{2}{\tilde{x}}^{T}P\tilde{x}+\frac{1}{2}tr\left({\tilde{W}}_{1}^{T}{\tilde{W}}_{1}\right)\text{.} \end{array}$$

From [15], we have ${\left[\partial g/\partial x\right]}_{x=\hat{x}}^{T}=\left[0,0,{\dot{U}}_{oc}\left(S\hat{O}C\right)\right]$ with $\alpha_{\min}\leq {\dot{U}}_{oc}\left(S\hat{O}C\right)\leq \alpha_{\max}$, where *α*_min_ and *α*_max_ are the minimum and maximum values of the change rate of the ${\dot{U}}_{oc}$ function, respectively. Then, the derivation of (12) gives

(13) $$\begin{array}{c} {\dot{V}}_{1}\leq \left.\frac{1}{2}{\dot{\tilde{x}}}^{T}\left[PA+A^{T}P-RMC-C^{T}{\left(RM\right)}^{T}\right]-2Q\right]\tilde{x}\\ +{\tilde{x}}^{T}PW_{1}^{T}\left(\sigma \left(x\right)-\sigma \left(\hat{x}\right)\right)+{\tilde{x}}^{T}P\cdot {\tilde{W}}_{1}\sigma \left(\hat{x}\right)+{\tilde{x}}^{T}P\varepsilon +\frac{1}{2}tr\left({\dot{\tilde{W}}}_{1}^{T}{\tilde{W}}_{1}+{\tilde{W}}_{1}^{T}{\dot{\tilde{W}}}_{1}\right)\text{,} \end{array}$$

where $M={\left[\begin{array}{ccc} m_{1}, & m_{2}, & m_{3} \end{array}\right]}^{T}\in {\mathbb{R}}^{3}$.

According to the unilateral Lipschitz condition [9], the following inequalities can be obtained:

(14) $$\begin{array}{c} {\tilde{x}}^{T}P\varepsilon \leq \frac{1}{2}{\left\|\tilde{x}\right\|}^{2}+\frac{1}{2}{\left\|P\right\|}^{2}\overset{3}{\underset{i=1}{\sum }}\varepsilon_{i}^{*2}\text{,} \end{array}$$

(15) $$\begin{array}{c} {\tilde{x}}^{T}PW_{1}^{*T}\left(\sigma \left(x\right)-\sigma \left(\hat{x}\right)\right)\leq {\left\|\tilde{x}\right\|}^{2}+{\left\|P\right\|}^{2}{\left\|W_{1}\right\|}^{2}\text{.} \end{array}$$

Taking (14) and (15) into (13), and considering tr(ab^*T*^)=tr(*b*^*T*^*a*)=*b*^*T*^*a*, we have

(16) $$\begin{array}{c} {\dot{V}}_{1}\leq \frac{1}{2}{\tilde{x}}^{T}\left[PA+A^{T}P-RMC-C^{T}{\left(RM\right)}^{T}-2Q\right]\tilde{x}+{\left\|\tilde{x}\right\|}^{2}+{\left\|P\right\|}^{2}{\left\|W_{1}\right\|}^{2}+\frac{1}{2}{\left\|\tilde{x}\right\|}^{2}\\ +\frac{1}{2}{\left\|P\right\|}^{2}\overset{3}{\underset{i=1}{\sum }}\varepsilon_{i}^{*2}+tr\left({\tilde{W}}_{1}^{T}\sigma \left(\hat{x}\right){\tilde{x}}^{T}P+{\tilde{W}}_{1}^{T}{\dot{\tilde{W}}}_{1}\right)\text{.} \end{array}$$

Based on [8], let PA+*A*^*T*^*P* − RMC − *C*^*T*^(RM)^*T*^ − 2*Q*=−Ψ, where $Q=\left[\begin{array}{ccc} 0 & 0 & 0\\ 0 & 0 & 0\\ 0 & 0 & \alpha_{\min}^{2} \end{array}\right]$; thus, (16) can be further written as

(17) $$\begin{array}{c} {\dot{V}}_{1}\leq -a_{0}{\left\|\tilde{x}\right\|}^{2}+\frac{1}{2}{\left\|P\right\|}^{2}{\left\|{\tilde{W}}_{1}\right\|}^{2}+D_{0}\text{,} \end{array}$$

where *a*_0_=*λ*_min_(*ψ*) − 3/2 and *D*_0_=‖*P*‖^2^‖*W*_1_‖^2^+1/2‖*P*‖^2^∑_*i*=1_^3^*ε*_*i*_^2^.

If $\hat{d}\left(x\right)⟶d\left(x\right)$, then the term $1/2{\left\|P\right\|}^{2}{\left\|{\tilde{W}}_{1}\right\|}^{2}+D_{0}$ in (17) can converge to zero. Moreover, by selecting the appropriate matrix *ψ*, *λ*_min_(*ψ*) can be relatively large. According to (17), the observation error can converge to a small neighborhood containing the origin.

### 3.2. Optimal Control Design Based on the Observer

#### 3.2.1. Online Policy Learning Algorithm

In this section, based on critic NN, we construct the policy learning law. Thus, system (8) can be rewritten as

(18) $$\begin{array}{c} \dot{\hat{x}}=F\left(\hat{x}\right)+Bu\text{,} \end{array}$$

where $F\left(x\right)=Ax+{\hat{W}}_{1}^{T}\sigma \left(x\right)+L{\left[\partial g/\partial x\right]}_{x=\hat{x}}^{T}\left(y-\hat{y}\right)$, and *L* is the Lyapunov function.

To realize the optimal control, we first define the cost function as\

(19) $$\begin{array}{c} V\left(\hat{x},u\right)=\int_{0}^{\infty }r\left(\hat{x},u\right)ds\text{.} \end{array}$$

With $r\left(\hat{x},u\right)={\hat{x}}^{T}Q_{s}\hat{x}+u^{T}R_{s}u$ being the utility function, *Q*_*s*_ ∈ *ℝ*^3×3^ and *R*_*s*_ ∈ *ℝ* are the weight matrices of proper dimension.

We define the Hamiltonian function of the optimal control problem and the optimal cost function as

(20) $$\begin{array}{c} H\left(\hat{x},u,\nabla V\left(\hat{x}\right)\right)=r\left(\hat{x},u\right)+{\left(\nabla V\left(\hat{x}\right)\right)}^{T}\left(F\left(\hat{x}\right)+Bu\right)\text{.} \end{array}$$

(21) $$\begin{array}{c} V^{*}\left(\hat{x}\right)=\min_{u}\int_{0}^{\infty }r\left(\hat{x},u\right)ds\text{.} \end{array}$$

The optimal cost function $V^{*}\left(\hat{x}\right)$ is the solution of the following HJB equation:

(22) $$\begin{array}{c} 0=\min_{u}H\left(\hat{x},u,\nabla V^{*}\left(\hat{x}\right)\right)\text{.} \end{array}$$

With ∇*V*^*∗*^(*x*)=*∂V*^*∗*^(*x*)/*∂x*, we can obtain this optimal control action as

(23) $$\begin{array}{c} u^{*}=-\frac{1}{2}R_{s}^{-1}B^{T}\nabla V^{*}\left(\hat{x}\right), \end{array}$$

and the HIB equation in terms of ∇*V*^*∗*^(*x*) as

(24) $$\begin{array}{c} 0={\hat{x}}^{T}Q_{s}\hat{x}+{\left(\nabla V^{*}\left(\hat{x}\right)\right)}^{T}F\left(\hat{x}\right)-\frac{1}{4}{\left(\nabla V^{*}\left(\hat{x}\right)\right)}^{T}BR_{s}^{-1}B^{T}\nabla V^{*}\left(\hat{x}\right), \end{array}$$

with *V*^*∗*^(0)=0.

To realize the policy learning, some iteration procedure can be given as follows:

(1) Select the small positive number *τ*. Set *i*=0 and *V*^(0)^=0, and then give an initial admissible control *u*^(0)^.
(2) Using the control *u*^(*i*)^, resolve
  (25) $$\begin{array}{c} 0=r\left(\hat{x},u\right)+{\left(\nabla^{i+1}V\left(\hat{x}\right)\right)}^{T}\left(F\left(\hat{x}\right)+Bu^{i}\right), \end{array}$$
with *V*^(*i*+1)^(0)=0.
(3) Update the control action using
  (26) $$\begin{array}{c} u^{\left(i+1\right)}=\frac{1}{2}R_{s}^{-1}B^{T}\nabla V^{\left(i+1\right)}\left(\hat{x}\right)\text{.} \end{array}$$
(4) If $\left\|V^{\left(i+1\right)}\left(\hat{x}\right)-V^{\left(i\right)}\left(\hat{x}\right)\right\|\leq \tau$, stop, then apply the optimal control; else, let *i*=*i*+1 and go back to (2).

This algorithm will be convergence to the optimal control and optimal cost function when *i*⟶*∞*. The convergence of this algorithm can be referred to [16, 17].

#### 3.2.2. NN Implementation

We assume the cost function $V\left(\hat{x}\right)$ is continuously differentiable. Then, we can use the NN reconstruct the $V\left(\hat{x}\right)$ as

(27) $$\begin{array}{c} V\left(\hat{x}\right)=W_{2}^{T}\sigma_{c}\left(\hat{x}\right)+\varepsilon_{c}\left(\hat{x}\right)\text{,} \end{array}$$

where *W*_2_ ∈ *ℝ*^*N*^ is the ideal NN weights, *σ*_*c*_(*x*) ∈ *ℝ*^*n*^ is the activation function, and $\varepsilon_{c}\left(\hat{x}\right)\in \mathbb{R}$ denotes the NN error. Then,

(28) $$\begin{array}{c} \nabla V\left(\hat{x}\right)={\left(\nabla \sigma_{c}\left(\hat{x}\right)\right)}^{T}W_{2}+\nabla \varepsilon_{c}\left(\hat{x}\right)\text{,} \end{array}$$

where $\nabla \sigma \left(\hat{x}\right)=\partial \sigma_{c}\left(\hat{x}\right)/\partial \hat{x}$ and $\nabla \varepsilon_{c}\left(\hat{x}\right)=\partial \varepsilon_{c}\left(\hat{x}\right)/\partial \hat{x}$ are the gradient of the activation function and NN error, respectively. According to (28), we can obtain the Lyapunov function as

(29) $$\begin{array}{c} 0=r\left(\hat{x},u\right)+\left(W_{2}^{T}\nabla \sigma_{c}\left(\hat{x}\right)+{\left(\nabla \varepsilon_{c}\left(\hat{x}\right)\right)}^{T}\right)\dot{\hat{x}}\text{.} \end{array}$$

**Assumption 3 .**

(see [12–14, 18]). If the NN weight *W*_2_, the NN error *ε*_*c*_, the gradient ∇*σ*_*c*_, and derivative ∇*ε*_*c*_ are bounded, then we can have *ε*_*c*_⟶0 and ∇*ε*_*c*_⟶0.

We define the estimation of (27) as

(30) $$\begin{array}{c} \hat{V}\left(\hat{x}\right)={\hat{W}}_{2}^{T}\sigma_{c}\left(\hat{x}\right)\text{.} \end{array}$$

Then, we have

(31) $$\begin{array}{c} \nabla \hat{V}\left(\hat{x}\right)={\left(\nabla \sigma_{c}\left(\hat{x}\right)\right)}^{T}{\hat{W}}_{c}. \end{array}$$

with $\nabla \hat{V}\left(\hat{x}\right)=\partial \hat{V}\left(\hat{x}\right)/\partial \hat{x}$. Thus, the estimated Hamiltonian function can be given as

(32) $$\begin{array}{c} H\left(\hat{x},u,{\hat{W}}_{2}\right)=r\left(\hat{x},u\right)+{\hat{W}}_{2}^{T}\nabla \sigma_{c}\left(\dot{x}\right)\dot{\hat{x}}=e_{c}\text{.} \end{array}$$

To minimize error (32), we construct the objective function *J*=(1/2)*e*_*c*_^*T*^*e*_*c*_, and then the descent algorithm can be designed as

(33) $$\begin{array}{c} {\dot{\hat{W}}}_{2}=-\alpha_{1}\left[\frac{\partial J}{\partial W}\right]=-\alpha_{1}\left[\frac{\partial e_{c}}{\partial W}\right], \end{array}$$

with *α*_1_ > 0 being the learning gain of the NN.

Based on (29), the Hamiltonian function can be rewritten as

(34) $$\begin{array}{c} H\left(\hat{x},u,W_{2}\right)=r\left(\hat{x},u\right)+W_{2}^{T}\nabla \sigma_{c}\left(\hat{x}\right)\dot{\hat{x}}=e_{h}\text{,} \end{array}$$

where $e_{h}=-{\left(\nabla \varepsilon_{c}\left(\hat{x}\right)\right)}^{T}\dot{\hat{x}}$ is the residual error.

Define $\phi =\nabla \sigma_{c}\left(\hat{x}\right)\dot{\hat{x}}$, if there is a positive constant *ϕ*_*M*_ such that ‖*ϕ*‖ ≤ *ϕ*_*M*_, and denote the weight estimation error ${\tilde{W}}_{2}=W_{2}-{\hat{W}}_{2}$, and then based on (32) and (34), we have $e_{h}-e_{c}={\tilde{W}}_{2}^{T}\phi$; thus, we have the dynamic of the weight estimation error as

(35) $$\begin{array}{c} {\dot{\tilde{W}}}_{2}=-{\dot{\hat{W}}}_{2}=\alpha_{1}\left(e_{h}-{\tilde{W}}_{2}^{T}\phi \right)\phi \text{.} \end{array}$$

The persistent excitation (PE) condition is required to tune the NN, guaranteeing ‖*ϕ*‖ ≥ *ϕ*_*m*_ with *ϕ*_*m*_ being the positive constant. To this end, a probing noise is inserted into the system to meet the PE.

In this case, the optimal control action can be given as

(36) $$\begin{array}{c} u^{*}=-\frac{1}{2}R_{s}^{-1}B^{T}\left({\left(\nabla \sigma \left(\hat{x}\right)\right)}^{T}W_{2}+\nabla \varepsilon_{c}\left(\hat{x}\right)\right)\text{,} \end{array}$$

and its estimation is

(37) $$\begin{array}{c} \hat{u}=-\frac{1}{2}R_{s}^{-1}B^{T}{\left(\nabla \sigma \left(\hat{x}\right)\right)}^{T}{\hat{W}}_{2}\text{.} \end{array}$$

**Figure 2:**
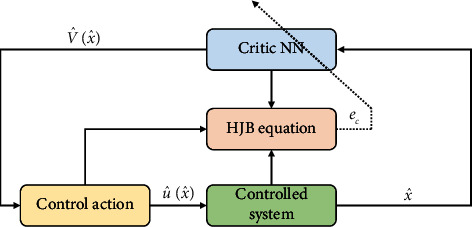
The structural diagram of the algorithm.

Equation (37) shows that using the trained critic network, the control policy can be derived directly; thus, the actor NN is removed in this paper. The structural diagram of the algorithm is given in Figure 2.

**Lemma 4 .**

For system (18), the adaptive law for the NN is provided by (33), and then the weight estimation error of NN is UUB.

**Proof**

Choose the Lyapunov function as $K\left(t\right)=\left(1/\alpha_{1}\right)tr\left({\tilde{W}}_{2}^{T}{\tilde{W}}_{2}\right)$. The time derivative of the Lyapunov function along the trajectory of error dynamics (35) is

(38) $$\begin{array}{c} \dot{K}\left(t\right)=\frac{2}{\alpha_{1}}tr\left({\tilde{W}}_{2}^{T}{\dot{\tilde{W}}}_{2}\right)=\frac{2}{\alpha_{1}}tr\left({\tilde{W}}_{2}^{T}\alpha_{1}\left(e_{h}-{\tilde{W}}_{2}^{T}\phi \right)\phi \right)\text{.} \end{array}$$

After doing some basic manipulations, we have

(39) $$\begin{array}{c} \dot{K}\left(t\right)\leq -\left(2-\alpha_{1}\right){\left\|{\tilde{W}}_{2}^{T}\phi \right\|}^{2}+\frac{1}{\alpha_{2}}e_{h}^{2}\text{.} \end{array}$$

Considering the Cauchy–Schwarz inequality and noticing the assumption ‖*ϕ*‖ ≤ *ϕ*_*M*_, we can conclude that $\dot{K}\left(t\right)<0$ as long as 1 < *α*_1_ < 2 and

(40) $$\begin{array}{c} \left\|{\tilde{W}}_{2}\right\|>\sqrt{\frac{e_{h}^{2}}{\alpha_{1}\left(2-\alpha_{1}\right)\phi_{M}^{2}}}\text{.} \end{array}$$

According to the Lyapunov theory, we obtain that the dynamics of the weight estimation error is UUB. The norm of the weight estimation error is bounded as well.

It is noted that the estimated weight ${\hat{W}}_{2}$ is optimal to *W*_2_, and this indicates that the solution $\hat{V}$ can be extracted from the estimated vector ${\hat{W}}_{2}$ given in (30). Thus, one can derive the actual control $\hat{u}=-1/2R_{s}^{-1}B^{T}{\left(\nabla \sigma \left(\hat{x}\right)\right)}^{T}{\hat{W}}_{2}$ for system (18) based on ${\hat{W}}_{2}$. As a consequence of Lemma 4, we can conclude that $\hat{u}$ will converge to the optimal control *u*^*∗*^, i.e., $\left\|\hat{u}-u^{*}\right\|⟶0$ such that the control system stability can be retained based on Lemma 4.

**Remark 5 .**

In this paper, an observer is designed using NN to online estimate the unknown state (SOC); then, based on the estimated state, we develop a policy learning algorithm to online resolve the optimal control of the battery. The proposed methods are different from our previous work, such as [18], where the system states are assumed to be known, and this limits the application of the optimal control algorithm in practice.

**Remark 6 .**

To realize the output-feedback control using the policy learning, the PE condition is required in this paper. As shown in [14, 17], to guarantee the PE condition, an alternative way is to insert an exploration noise into the system for the first two seconds [17].

## 4. Simulation Results

For the second-order RC equivalent model of power battery, the effectiveness of the optimal control theory in this paper is verified by simulation based on Matlab. The values of resistance, capacitance, and battery capacity in the second-order RC equivalent model (5) are as follows: *R*_0_=10.822mΩ, *R*_1_=3.103mΩ, *R*_2_=2.611mΩ, *C*_1_=8.4379kF, *C*_2_=91.401kF, and *Q*_*n*_=45A · h.

Let *M*=*I*, then we can obtain *P* and *L* as

(41) $$\begin{array}{c} P=\left[\begin{array}{ccc} 14.1250 & 0 & 19.6371\\ 0 & 128.7451 & 178.9860\\ 19.6371 & 178.9860 & 0 \end{array}\right]\text{,}\\ L=\left[\begin{array}{ccc} 0.0638 & -0.007 & 0.005\\ -0.007 & 0.0008 & 0.005\\ 0.005 & 0.005 & -0.0036 \end{array}\right]\text{.} \end{array}$$

Given the design parameters in learning law (33) as *α*_1_=0.1 and the initial values as *x*_1_(0)=0.1, *x*_2_(0)=0.2, *x*_3_(0)=1, ${\hat{x}}_{1}\left(0\right)=0.01,{\hat{x}}_{2}\left(0\right)=0,{\hat{x}}_{3}\left(0\right)=0.99$, and ${\hat{W}}_{2}=\left[0.3909\;0.5812\;1.0576\;0.1\;0.2\;1\right]$, we design the regressor of the critic NN as *σ*(*x*)=[*x*_1_^2^, *x*_1_*x*_2_, *x*_1_*x*_3_, *x*_2_^2^, *x*_2_*x*_3_, *x*_3_^2^]^*T*^.

We aim at obtaining an optimal control policy that can stabilize system (18). For system (18), we need to find a feedback control policy that minimizes the cost function.

(42) $$\begin{array}{c} V\left(\hat{x},u\right)=\int_{0}^{\infty }\left({\hat{x}}^{T}Q_{s}\hat{x}+u^{T}R_{s}u\right)ds, \end{array}$$

with *Q*_*s*_=*I* and *R*_*s*_=2*I*. We adopt the online policy iteration algorithm to tackle the optimal control problem, where a critic network is constructed to approximate the cost function. During the implementation process of the policy learning algorithm, we introduce the noise to meet the PE condition. The exponentially decreasing probing noise and sinusoidal signals with different frequencies are used. They are introduced into the control input and thus affect the system states.

**Figure 3:**
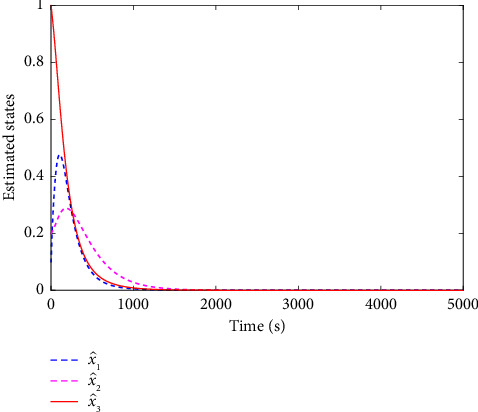
Estimated system states.

**Figure 4:**
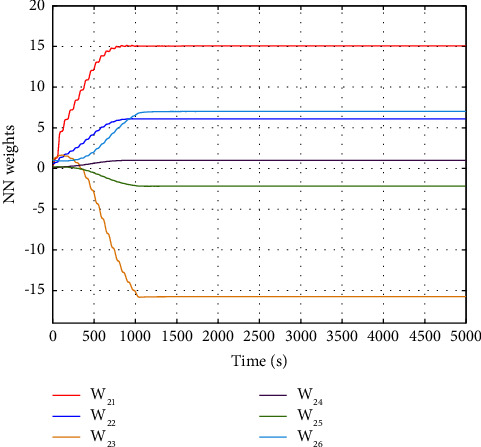
NN weights.

**Figure 5:**
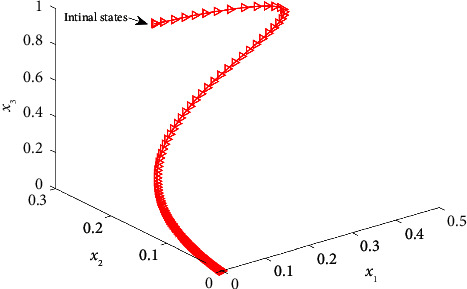
System trajectory.

**Figure 6:**
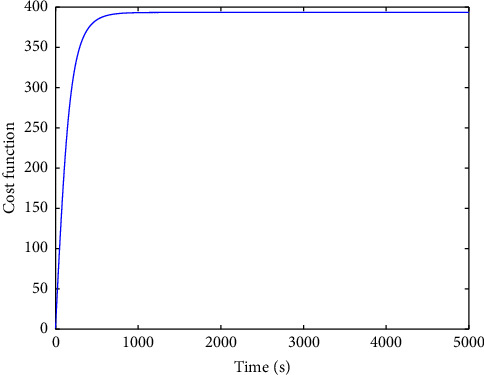
Cost function.

**Figure 7:**
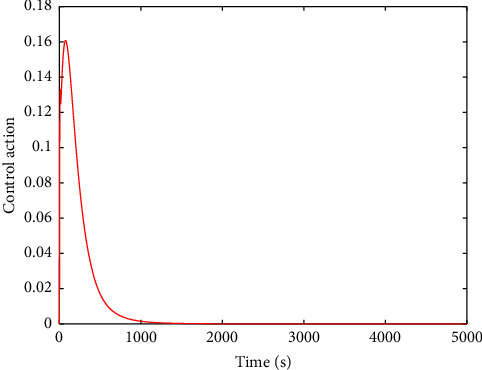
Control action *u*.

The evolution of the state trajectory is depicted in Figure 3, and this can be used to further design the optimal controller for the proposed system.  Figure 4 gives the good estimated weights, where we have that the convergence of the weight has occurred after 1000 s. Then, the probing signal is turned off. This good convergence of the NN weights can ensure the stability of the controlled system, which can be found in Figure 5. Figure 5 is the controller system trajectory with the designed optimal controller. We see that the state converge to zero after the probing noise is turned off.  Figure 6 shows the cost of the system under which is smooth, and this indicates that the designed controller is effective. The control action is given in Figure 7, which is bounded. This further shows Lemma 4 is true.

**Figure 8:**
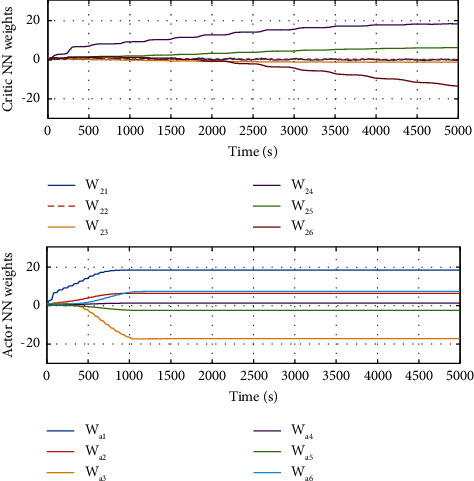
NN weights.

**Figure 9:**
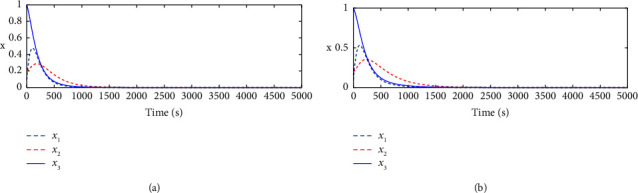
System state *x* (a) using the proposed method and (b) the method proposed in [19, 20].

To show the improved performance of the proposed single critic NN-based ADP for solving the derived optimal control problem, a critic-actor NN-based online learning method [19] is also used for comparison. Moreover, in this comparison, we add the robustness verification of the proposed method. To this end, we set the nonlinear term *d*(*x*)=0.5 sin(*x*_1_). The profiles of the critic NN and actor NN weights can be found in Figure 8 and the corresponding control performances are given in Figure 9. Compared with Figures 9(a) and 9(b), it is clear that the proposed single critic NN-based can achieve faster transient state convergence even if there is a nonlinear term.

Generally, the modeling accuracy and control structure will influence the control performance of the closed-loop control systems. In this paper, the main factors affecting the control performance are the modeling uncertainties of the system and the convergence performance of critic NN weights. Moreover, better convergence of critic NN weights, i.e., faster convergence speed can help to achieve better control performance. In this respect, different choices of critic NN parameters and structure will affect the convergence of critic NN weights and the control performance. Hence, proper selection of NN parameters and structure, such as the initial value of weights, learning gain, and regressor structure, is helpful to further improve the control response.

## 5. Conclusion

For the second-order RC equivalent nonlinear system of power battery, the unknown uncertainty of the system is approximated by NN, and a time-varying gain nonlinear state observer is designed to solve the problem that the resistance capacitance voltage and charge (SOC) of the battery cannot be measured. Then, to realize the optimal control, a policy learning-based online algorithm is designed, where only the critic NN is required, and the actor NN widely used in most design of the optimal control methods is removed. Finally, the effectiveness of the optimal control theory is verified by simulation.

## Data Availability

The data used to support the findings of this study are available upon request from the corresponding author.
